# Can We Predict Interface Dipoles Based on Molecular Properties?

**DOI:** 10.1021/acsomega.1c05092

**Published:** 2021-11-14

**Authors:** Johannes J. Cartus, Andreas Jeindl, Oliver T. Hofmann

**Affiliations:** Institute of Solid State Physics, Graz University of Technology, Petersgasse 16/II, 8010 Graz, Austria

## Abstract

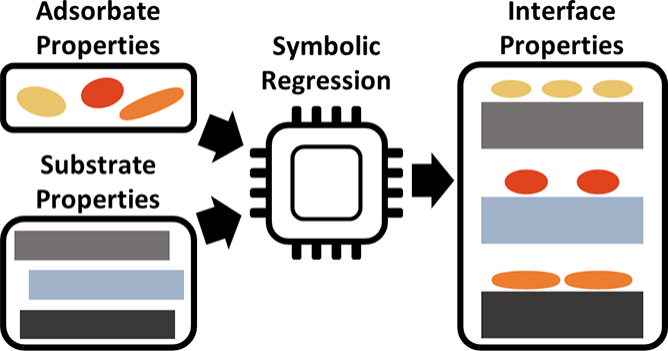

We apply high-throughput density functional theory calculations and symbolic regression to hybrid inorganic/organic interfaces with the intent to extract physically meaningful correlations between the adsorption-induced work function modifications and the properties of the constituents. We separately investigate two cases: (1) hypothetical, free-standing self-assembled monolayers with a large intrinsic dipole moment and (2) metal–organic interfaces with a large charge-transfer-induced dipole. For the former, we find, without notable prior assumptions, the Topping model, as expected from the literature. For the latter, highly accurate correlations are found, which are, however, clearly unphysical.

## Introduction

The level alignment of metal–organic interfaces has been subject to much attention from both fundamental^1−4^ and engineering research, especially in the context of organic electronics.^5−8^ Suboptimal choices in the design of interface materials can lead to poor device performance, for example, because of electrical resistances caused by large charge-injection barriers.^5^ However, these injection barriers, which depend on the offset between the metal’s Fermi energy and the molecular levels,^2^ can be optimized by adsorbing a so-called charge-injection layer onto the metal. These layers change the level alignment because of the emergence of an adsorption-induced potential jump^1−4^ (often termed “interface dipole”), ΔΦ.

Currently, ΔΦ must be determined separately for every substrate/adsorbate combination, either experimentally or via first-principles calculations, but both options are expensive and laborious.^9^ A prediction, or at least a solid estimate, of ΔΦ based solely on the properties of the isolated adsorbate and substrate would significantly speed up the optimization process for inorganic/organic interfaces. However, although there are several (often conflicting) models that relate molecular properties to ΔΦ (such as the induced density of interface states model,^10−13^ the integer charge-transfer model,^14−16^ or pinning on the lowest unoccupied molecular orbital (LUMO)^17−21^), an explicit expression describing the interface dipole via the properties of the constituents is yet to be put forth. In fact, it is not yet clear whether such an expression can be formulated based solely on the properties of the interface constituents at all.

In this work, we attempt to extract an analytic expression by a combination of high-throughput first-principles calculations and symbolic regression. In its most simple formulation, symbolic regression takes a number of input properties (e.g., the molecular dipole moment, the ionization energy, etc.; see below) and combines them via mathematical operators (e.g., multiplication, exponentiation, etc.) into more complex equations (i.e., analytic models). These expressions are then fitted against a target quantity (e.g., ΔΦ). Ideally, the best-fitting models correspond to the “natural laws” that govern the physics underlying the data.^22,23^ This approach can be seen as a one-dimensional variant of the sure independence screening sparsifying operator (SISSO) method^24^ (see the Supporting Information). However, the complexity from additional SISSO dimensions is not necessarily required (or helpful) for detecting physical relationships (see the Supporting Information).

When studying the interface-dipole-induced work function change, it has become customary to dissect it into two components:^18,25^ the contribution that arises from the bonding to the substrate, ΔΦ^Bond^, and the jump of the electrostatic potential that would be induced by the adsorbate alone, ΔΦ^Mol^:
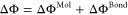
1

Because it is difficult, if not impossible, for symbolic regression to identify two different effects of similar magnitude (for explanation see the Supporting Information), here, we aim at obtaining analytical models for ΔΦ^Mol^ and ΔΦ^Bond^ separately.

## Results and Discussion

### Adsorbate Dipole

**Figure 1:**
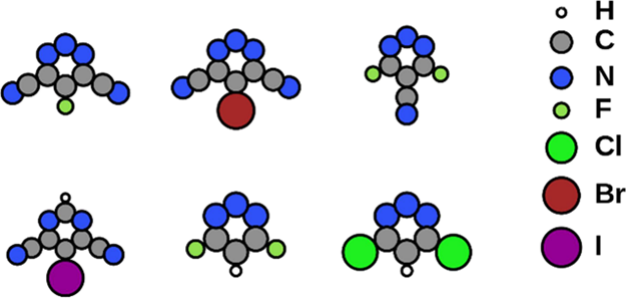
The six heteroaromatic molecules we used to build free-standing self-assembled monolayers.

Starting with the adsorbate-dipole-induced potential jump ΔΦ^Mol^, we design several planar heteroaromatics with substantial in-plane dipole moments through specific substitution of halogens and nitrogen. From these, we select the six molecules with the largest electron affinity (shown in Figure 1), which will be useful for the investigation of ΔΦ^Bond^ later in this work. We then create hypothetical, free-standing self-assembled monolayers (i.e., without substrates) by placing the molecules, with their intrinsic dipole moments aligned in the *z*-direction, in various unit cells with various side lengths (12.5–30 Å) and angles (45, 60, 75, and 90°). The combination of molecules and unit cells yields 360 different systems. An example is depicted in the Supporting Information. For these systems, we obtain ΔΦ^Mol^ by performing dispersion-corrected density functional theory (DFT) calculations with the Fritz Haber Institute ab initio materials simulations package (FHI-aims)^26^ using the Perdew–Burke–Ernzerhof (PBE) exchange-correction functional^27^ and a dipole correction^28^ (further details are given in the Methods section).

To extract analytic models for ΔΦ^Mol^, our symbolic regression algorithm takes various properties of the interface or the isolated molecules in the gas phase as input. We then combine these input properties via mathematical operations to build analytical expressions. To keep the number of possible expressions tractable, we adhere to the following protocol: in the first step, we exhaustively create products of up to three input parameters and their reciprocals, that is, we create expressions of the form *F*(*x*, *y*, *z*) = *x^a^y^b^z^c^* with three different input parameters *x*, *y*, *z* and exponents *a*, *b*, *c* ∈ {–1,0,1}. In the second step, we create additional expressions by applying the nonlinear mapping *F*′(*F_i_*, *F_j_*) = *F_i_*/(*F_j_* + 1) to all possible pairs of expressions, with the restriction that the factors *x*, *y*, *z* in *F_i_* and *F_j_* can only differ in a single input parameter. Finally, all created expressions are evaluated using the input parameter values from the systems in the data set. For each analytical expression, a linear fit against ΔΦ^Mol^ is performed, and the best-performing fit (in terms of its root-mean-square-error, RMSE) is reported.

Because the resulting set of analytical expressions grows very fast with the number of input parameters, a thoughtful selection is required. Here, we use the following properties for the isolated molecules in the gas phase as input parameters: the orbital energy of the highest occupied molecular orbital (ε_HOMO_) and of the LUMO (ε_LUMO_), the ionization potential (IP) and the electron affinity (EA) via the ΔSCF approach (see the Methods section), the molecular dipole moment μ, and the molecular polarizability α along the direction of the dipole moment. In addition, we provide the lengths of the unit cell vectors (*a*,*b*), the minimum distance between two atoms (*d*_min_), and *C*_Σ_, the infinite sum of cubed reciprocal distances from a dipole to all its neighbors, as geometry-dependent input parameters. The latter often appears in the electrostatic description of collective electric fields of dipoles.^29^ A compilation of all used parameters is provided in Table 1.

**Table 1:** Compilation of Input Parameters Used to Construct the Candidate Analytical Expressions for ΔΦ^Mol^

name	description
*a*,*b*	unit cell side lengths
*d*_min_	minimum distance between atoms of periodic replicas of adsorbate molecules
ρ	dipole density (number of molecules per area)
*C*_Σ_	infinite sum of cubed reciprocal distances *r*_i_ from a molecule to all its neighbors: *C*_Σ_ = ∑_*i*_*r*_*i*_^–3^.
ε_HOMO_, ε_LUMO_	orbital energies of the isolated molecule
IP, EA	vertical IP and EA of the isolated molecule
μ_*z*_	*z*-component of the single molecule dipole moment in vacuum
α_zz_	molecular polarizability along the direction of the dipole moment

For our systems, the expression with the lowest RMSE (3.3 meV) we found is shown in eq 2.
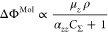
2

**Figure 2:**
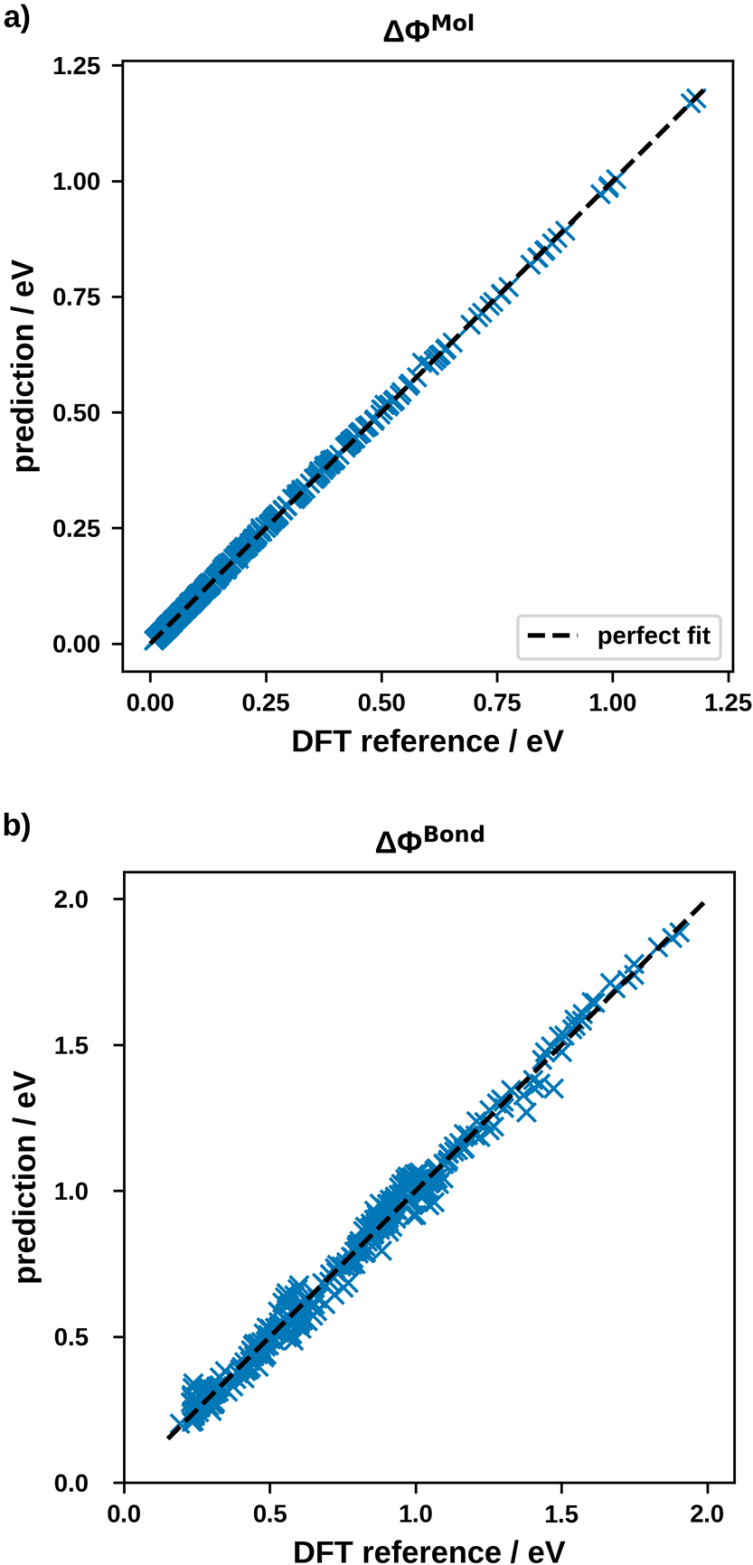
Prediction of (a) ΔΦ^Mol^ and (b) ΔΦ^Bond^ versus the DFT-calculated reference data. The dashed line marks a hypothetical perfect fit.

The excellent agreement between the prediction of ΔΦ^Mol^ via eq 2 and the “true” values originally obtained by DFT is displayed in Figure 2a. Eq 2 is exactly the Topping model, which is expected from classical electrostatics^29−31^ and was previously suggested by numerous other theoretical^32−34^ and experimental^31,35^ studies.

While finding the Topping model from our data shows the validity of our approach, it is important to emphasize that this success is by no means guaranteed. Obviously, we could only find eq 2 because we allowed for the nonlinear mathematical operation and because we provided *C*_Σ_ as input parameter. Neither of these would necessarily be intuitive, but without either of these, we would only obtain physically incorrect solutions. Interestingly, when including additional systems that are too densely packed (i.e., when the point-dipole approximation underlying the Topping model^29^ starts to break down), some of these unphysical models exhibit even lower RMSE values (i.e., perform even better) than the physically correct expression. Nevertheless, under the correct boundary conditions, the “correct” physical picture can be accurately obtained from our data.

### Bond Dipole

As the second step, we turn to ΔΦ^Bond^. This term contains all effects of ΔΦ arising from the interaction of the adsorbate with the substrate, such as charge transfer, Pauli pushback, formation of covalent bonds, and so forth.^1−4^ In this work, we focus on charge transfer only because it is (a) relatively straightforward to separate from the other effects and because (b) one would expect that the molecular properties that govern can be easily tuned via chemical modifications of the adsorbate.

To increase the diversity in our data set (compared to the previous section), here, we use a total of 28 different heteroaromatic molecules, consisting of the 6 molecules used before and 22 additional molecules that are based on naphthalene as a backbone to allow for more varied molecular properties (for details, see the Supporting Information). A challenge when considering ΔΦ^Bond^ is that it is known to depend strongly on the geometry the adsorbate assumes. For instance, whether a molecule adsorbs flat-lying or upright-standing can change ΔΦ^Bond^ by more than 1 eV^36−38^ because of the associated change in the molecule’s ionization energies.^39^ Similarly, ΔΦ^Bond^ is strongly affected by the molecular coverage.^33,40,41^ However, on metal substrates, most molecules adsorb in an approximately flat-lying geometry,^42^ often slightly bent^17,43,44^ or with a small tilt.^44−47^ To simplify the physics to be described, here, we assume hypothetical geometries in which all molecules remain perfectly flat. This has the further advantage that their molecular dipole moment is parallel to the xy-plane, such that it does not contribute to ΔΦ. To remove any coverage-dependent effects, we use the same supercell, that is, adsorbate density, throughout. This geometry is an Ag(111) surface slab with five layers and a surface area of 5 × 5 Ag atoms. The large supercell ensures that there is only very little interaction between adjacent molecules. To focus on charge-transfer alone, we use hypothetical interfaces where the adsorption height is sufficiently large to inhibit wave-function overlap between the substrate and the adsorbate, thereby switching off any contributions from Pauli pushback or covalent bonds. In practice, we adsorb molecules at distances between 7 and 100 Å above metal slabs made of Ag, Al, In, Mg, and Na. In total, this results in 323 different interface systems to evaluate our expressions on.

Also, here, we must select suitable input parameters for the symbolic regression approach. We start by taking the ones suggested by the various models in the literature:

- The induced density of interface states implies a relation to the density of states at the Fermi level (a substrate property),^10,11^ which we also include here.
- The so-called integer charge-transfer model postulates that charge transfer occurs via polaronic levels.^14−16^ While polarons are a crystal quantity and their relation to purely molecular properties is not straightforward, here, we incorporate them approximately as the molecule’s vertical EA and the relaxation energy *E*_relax_. The latter refers to the difference between the energy of the singly charged molecule in the geometry of the neutral molecule and when it is fully optimized, that is, its internal reorganization energy. Because we are interested in work function changes, we use EA relative to the substrates’ work function.
- Many previous theoretical calculations imply that ΔΦ^Bond^ is determined by the difference between the frontier orbital energies and the Fermi energy.^18,19^

We also add the polarizability of the molecule perpendicular to the aromatic plane, α_*zz*_, and the HOMO–LUMO gap because it is a common measure for the reactivity of a molecule.^48^ Furthermore, it is conceivable that image-charge effects play a role. As geometric properties, we therefore include the height of the molecule above the substrate’s image plane position (for details, see the Supporting Information) and above the topmost layer as input parameters. A comprehensive compilation of input parameters is given in Table 2. Otherwise, we construct expressions as we did for ΔΦ^Mol^, that is, we build products *F* of up to three input parameters (or their reciprocals) and create additional expressions via the nonlinear mapping *F*′ = *F_i_*/(*F_j_* + 1) using the same conditions as above.

**Table 2:** Compilation of Input Parameters Used to Construct the Candidate Analytical Expressions for ΔΦ^Bond^

name	description
ε_LUMO_ – *E*_F_	difference of the LUMO of the adsorbate and the Fermi energy of the substrate.
ε_HOMO_ – *E*_F_	difference of the HOMO of the adsorbate and the Fermi energy of the substrate.
EA – Φ	difference of the EA of the adsorbate and the work function of the substrate.
IP – Φ	difference of the IP of the adsorbate and the work function of the substrate.
*E*_relax_	relaxation energy of the adsorbate (see the main text).
DOS(*E*_F_)	density of states of the pristine substrate at the Fermi energy.
*h* – *z*_im_	adsorption height of the molecule w.r.t the image plane position.
*h*	adsorption height of the molecule w.r.t the uppermost substrate layer.
ε_LUMO_ – ε_HOMO_	HOMO–LUMO gap.
α_*zz*_	*zz*-component of the polarizability tensor of the adsorbate.

For the bond dipole, we find the best-performing expression to be as follows:
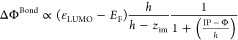
3

With an RMSE of 38 meV, it performs reasonably well (see Figure 2b), even if the RMSE is one order of magnitude worse than the Topping model for the ΔΦ^Mol^.

Inspection of eq 3 reveals that it is almost exclusively dominated by the term (ε_LUMO_ – *E*_F_). The second term, *h/(h – z*_im)_, is always larger than but close to 1 (between 1.0 and 1.5), because *h* ≫ *z*_im_ for the majority of systems in our data set. Conversely, the third term is also always close to one, but smaller (between 0.5 and 1.0), because within the units used here, the numerical value for IP-Φ/*h* is small for all systems. In fact, it must be stressed that this third term cannot carry any significance beyond numerics, as the physical dimensions of the featured parameters do not match. We allow for such “numeric terms” to achieve a greater variety in the generated expressions. Because the second and third term also counteract each other (both get closer to 1 as *h* increases), the ΔΦ^Bond^ values predicted using eq 3 scatter only very little around ε_LUMO_ – *E*_F_. This may, in principle, indicate that our computations yield noisy results for ΔΦ^Bond^. However, our self-consistent field (SCF) procedure converged ΔΦ to 10^–4^ eV (see the Methods section), that is, much too tight to allow for noise of this magnitude. Furthermore, we note that ε_LUMO_ is an auxiliary quantity from DFT. There is some debate about when orbital energies correspond to observables (e.g., photoemission resonances).^49^ However, they always do depend on the chosen exchange-correlation functional, that is, the chosen theoretical method. In an earlier work, we have shown that there is no direct proportionality between (ε_LUMO_ – *E*_F_) and ΔΦ^Bond^ when going, for example, from semilocal to hybrid functionals.^19^ In other words, in salient contrast to the Topping model found in eq 2, eq 3 fails to extract the physics that governs the charge transfer at the interface (rather, it merely shows an excellent correlation).

There are multiple possible reasons for this. In principle, it would be conceivable that some of our data are faulty. However, we can readily extract other physical relationships (see the Supporting Information), which attest to the fidelity of our results. Another possible explanation would be that we do not include the correct input parameters and mathematical operations or that we do not allow for sufficiently complex expressions. However, also various other additional input parameters and more varied exponents and (nonlinear) functions fail to yield physically meaningful results (see the Supporting Information). While this is no proof that we just did not include the “right” ingredients (such a proof is fundamentally impossible), it seems unlikely that the correct relation is an expression that is even more complex than what we already found for eq 3. Finally, we must face the hypothesis that our data, being synthetic, computed data with an approximate theory, just do not reproduce the underlying physics with sufficient accuracy.

Indeed, the PBE functional is known to have certain issues when describing charge-transfer systems. For example, when dissociating H_2_^+^ (i.e., placing the two H-cores far away from each other), it yields the unphysical solution of two protons with half an electron each, instead of a neutral H and a positively charged H^+^ atom. Also here, we find that the molecules, even far above the surface (and thus completely unhybridized with it), are fractionally charged. In principle, a physically more correct solution could be a mixture of charged and uncharged molecules, that is, integer charge transfer. This could only be obtained by employing large supercells in combination with specifically tuned hybrid functionals.^19^ However, the optimal functional would have to be determined separately for each system,^50^ which incurs computational costs that are presently intractable. At the same time, it is not clear whether this would even solve the issue: because ΔΦ depends on the average amount of charge transferred per area, not its distribution, a computation with hybrid functionals may not yield more accurate values. A further related problem is the self-interaction error of PBE. This effect not only causes the well-known underestimation of the band gap but it also makes the energies of the orbitals (including the LUMO) dependent on their occupation. This shift of the orbital energy may be superimposed to the shift of the orbital energy induced by ΔΦ^Bond^, making it impossible for symbolic regression to extract either effect.

## Conclusions

In summary, we attempted to extract the physics that govern the formation of interface dipoles at inorganic-organic interfaces. To that aim, we computed large data sets using semilocal DFT and applied symbolic regression to obtain functional relationships between the properties of the molecules (and the substrate) and ΔΦ. The approach was successful for the contribution of the molecular dipole, yielding the well-known Topping model. Conversely, for the charge-transfer contribution, we obtained a clearly unphysical result that depends on a DFT quantity rather than a molecular property. We tentatively attribute the failure to extract a clear physical relationship to the shortcomings of the underlying method (PBE). Despite the generally outstanding performance of dispersion-corrected PBE calculations for interfaces,^51−54^ this advises that caution should be taken when computing interfaces with a notable charge-transfer character.

Moreover, the difficulty to extract the relevant physics for charge transfer even with a very large data set and an extensive combination of molecular and substrate parameters shows that the design of inorganic–organic interfaces with a predefined level alignment is nontrivial and will continue to be so. Even when minimizing the impact of the adsorbate geometry (which is extremely difficult to predict in the first place), and when simplifying the problem by avoiding quantum mechanical interactions (such as Pauli pushback and covalent bonds) as much as possible, we can only extract empirical correlations so far. For a comprehensive understanding and description of all effects at the interface, evidently much larger, more sophisticated data sets are still needed.

## Methods

All DFT calculations mentioned in the study were performed using FHI aims.^26^ This code allows employing both open and periodic boundary conditions, that is, individual molecules and interfaces can be treated on the same footing. For all systems, we used tight basis sets and numerical defaults as shipped with release 201103. The PBE^27^ exchange-correlation functional was used together with the vdW-TS^55^ dispersion correction.

To obtain the properties of the individual molecules, we performed calculations with open boundary conditions. The geometry of the (charge-neutral) molecules was fully relaxed until the remaining forces on each atom fell below 0.01 eV/Å. From the optimized geometry, we extracted the orbital energies of the HOMO and LUMO, the molecular dipole moment, and the polarizability (via density functional perturbation theory^56^). Furthermore, we calculated the vertical IP and EA using the so-called ΔSCF-approach.^57,58^ There, these energies are given as the energy difference between the singly charged and the uncharged molecule while keeping the geometry of the neutral molecule. The singly charged molecules are calculated spin-polarized (which is not necessary for the neutral molecules). We employed a Gaussian occupation scheme with a broadening of 0.01 eV.

All other calculations (free-standing monolayer, metal–organic interfaces, and bare metals) were performed with periodic boundary conditions. Calculations for the bare substrate as well as for metal–organic interfaces were performed using five metal layers. We employed a repeated slab approach to emulate two-dimensional periodicity. The unit cell heights were chosen so that the vacuum amounts to at least 50 Å. To electrostatically decouple the periodic replica in the *z*-direction, we used a dipole correction.^28^ The SCF algorithm was repeated until total energies in subsequent iterations differed by less than 10^–5^ eV and electron densities differed by less than 10^–3^ electrons. Furthermore, we ensured for all calculations that the change in the work function is converged to better than 10^–4^ eV between subsequent SCF iterations, as suggested by best practices.^54^ We employed a generalized Monkhorst–Pack k-point grid^59,60^ that corresponds to 50 × 50 × 1 k-points for the primitive substrate cells. Furthermore, a Gaussian occupation scheme with a broadening of 0.1 eV was used.

## Supporting Information Available

Visualization of systems in the data set, comparison of our method and SISSO, finding concurrent effects, listing of naphthalene-derived adsorbates, calculation of image plane positions, data set sanity checks and analysis of alternative input parameters, and nonlinear mappings for expression generation (PDF)

## Notes

All DFT calculations are available in the NOMAD repository (dx.doi.org/10.17172/NOMAD/2021.10.27-1).
