# Supplementary material for: Can We Predict Interface Dipoles Based on Molecular Properties?
Source: ACS Omega. 2021 Nov 14;6(47):32270–6. doi: 10.1021/acsomega.1c05092 (PMC8638305; doi:10.1021/acsomega.1c05092)
Supplement: Supplementary file 1 — ao1c05092_si_001.pdf [file ao1c05092_si_001.pdf]

Supporting Information to “Can We Predict Interface Dipoles Based on Molecular Properties?”

Johannes J. Cartus, Andreas Jeindl, and Oliver T. Hofmann*

Institute of Solid State Physics, Graz University of Technology, Petersgasse 16/II, 8010 Graz, Austria

Visualization of the Systems in Our Data Set

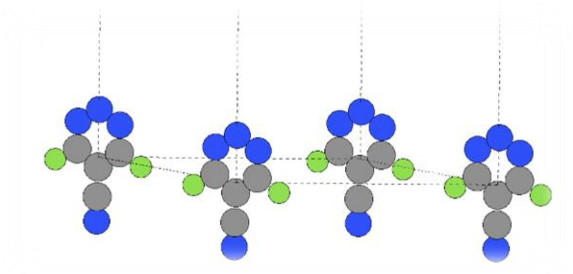

Figure S1: *Visualization of one of our free-standing monolayer systems: $C_4N_4F_2$ in a hexagonal unit cell with a side length of 12.5 Å. The dashed lines represent the unit cell.*

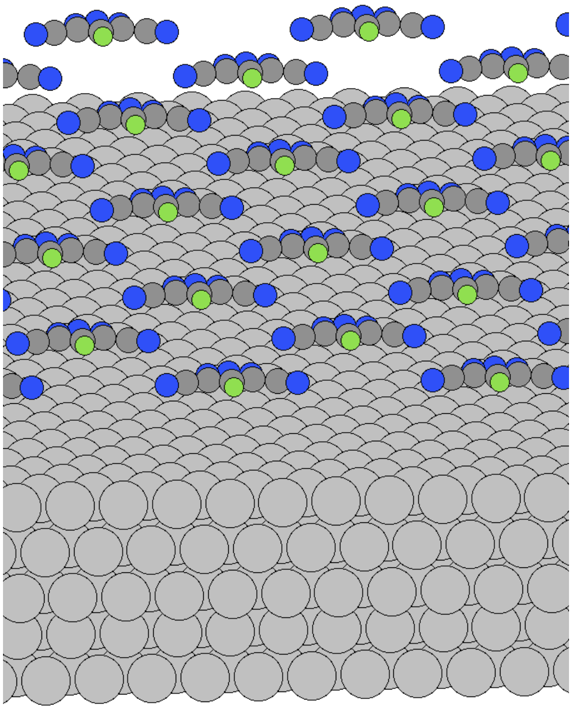

Figure S2: *Visualization of one of the interface systems in our data set. C_5H_5F on an Ag(111) slab with an adsorption height of 7.5 Å.*

Our Method vs SISSO

SISSO[1] (Sure Independence Screening Sparsifying Operator) works in an iterative, two-stage process to find the expression that best describes a target quantity. Expressions are generated and evaluated as they are in our approach; see main manuscript. The first stage preselects expressions based on correlation with the target and the second stage builds linear models out of the preselected expressions. In the first iteration it finds the single best analytic expression to describe the target as a linear model. This is equivalent to our method. In the next iteration the residual of the previous linear model is calculated, and expressions are preselected based on correlation with the residual. Again, linear regression is used to produce a linear model describing the target (now with 2 terms). This is repeated until the final iteration is reached (the linear model now has as many terms as iterations were performed). The number of iterations/terms is a user specified parameter and called "dimension of the descriptor".

The preselection based on the residual of previous iterations is what makes the method impractical for our uses. Say the target physics we want to describe is given by the sum of two terms. Intuitively, it seems logical to aim for a two-dimensional descriptor. However, as neither of the individual terms correlates well with the target data, neither of them is preselected in the first stage of the SISSO iterations. This makes it impossible to find a two-dimensional descriptor describing the data. Conversely, if the sum of the two terms is considered as an expression directly, it will most probably be found because the expression will correlate well with the target. This corresponds to a one-dimensional descriptor for the SISSO method, thus showing that additional dimensions are not additionally useful.

Finding Concurrent Effects at the Same Time

Our method relies heavily on sensible expressions being better correlated with the target than other, unphysical expressions. The threshold for necessary correlation is very high, as is also demonstrated by the results in the main manuscript. There, the best performing expressions show Pearson correlation coefficients[2] greater than 0.95; this includes also clearly unphysical expressions. Sensible expressions are thus most easily found if the corresponding physics is well represented in the data. However, when there are multiple, concurrent physical effects present in the data it obviously becomes harder to capture singular effects.

We demonstrate this with a 1-D example: assuming the physics of a problem at hand is given by a known 1-D function, one can see how well the known function correlates with the available data when we introduce a secondary effect as perturbation. Let the physics of interest be described by a sine function of an independent variable t. Let an additional, concurrent effect be described by a cosine. The available data shall then be given by

$$F_\alpha(t) = (1-\alpha)\sin(t) + \alpha\cos(t). \quad (1)$$

α is the mixing parameter which determines how much of the concurrent effect is mixed into the target (i.e. how strong the perturbation is compared to the main effect). For $t \in [0, 10]$ we have plotted the functions below in Figure S3.

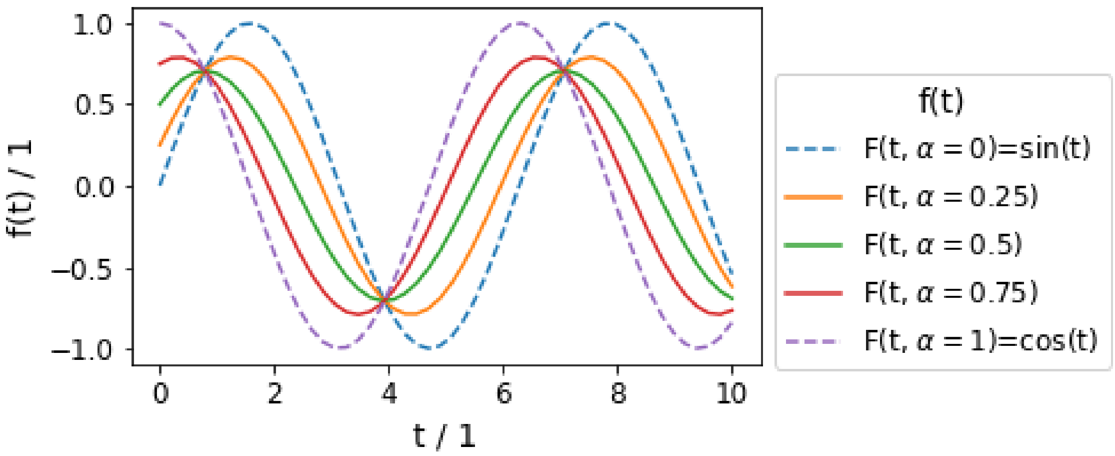

Figure S3: *Various functions f(t) of the independent variable t. The values of $F_\alpha(t)$ represent available data used to calculate correlation coefficients.*

Even though sine, cosine and F_α are very similar in their functional form, the correlation $\sin(t)/F_\alpha(t)$, measured via Pearson correlation coefficient ρ, decreases with increasing α, until it vanishes (Figure S4).

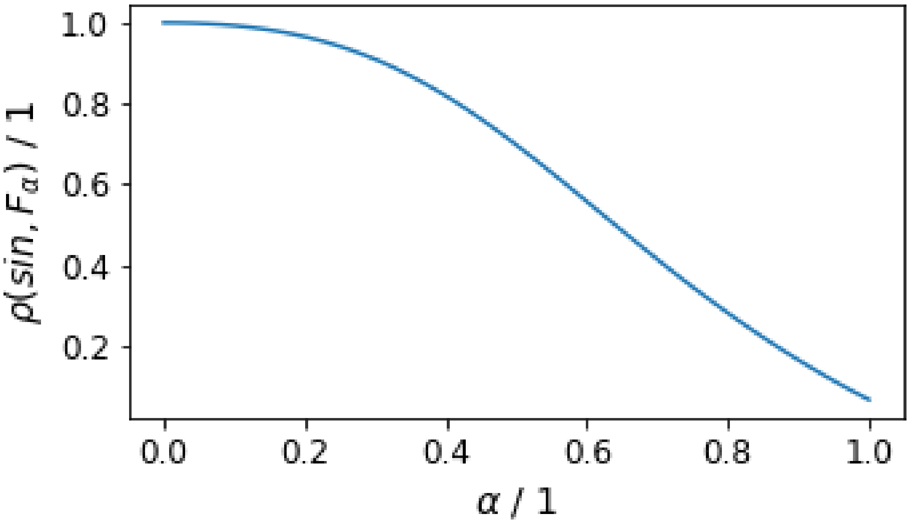

Figure S4: *Correlation of sin(t) with $F_\alpha(t)$ for different values of α, measured with Pearson correlation coefficient ρ for t ϵ [0, 10].*

This shows very nicely how a concurrent effect can interfere with detecting physics even in this very simplified example. In reality, one faces multiple concurrent effects simultaneously, which depend on multiple independent variables in addition to noise and systematic errors from the method used for measurement/computation.

Naphthalene-Derived Heteroaromatic Molecules

In addition to the 6 benzene-derived heteroaromatic molecules (Figure 1 in the main manuscript) we used the following 22 naphthalene-based molecules (Figure S5) as adsorbates in the metal-organic interfaces we study.

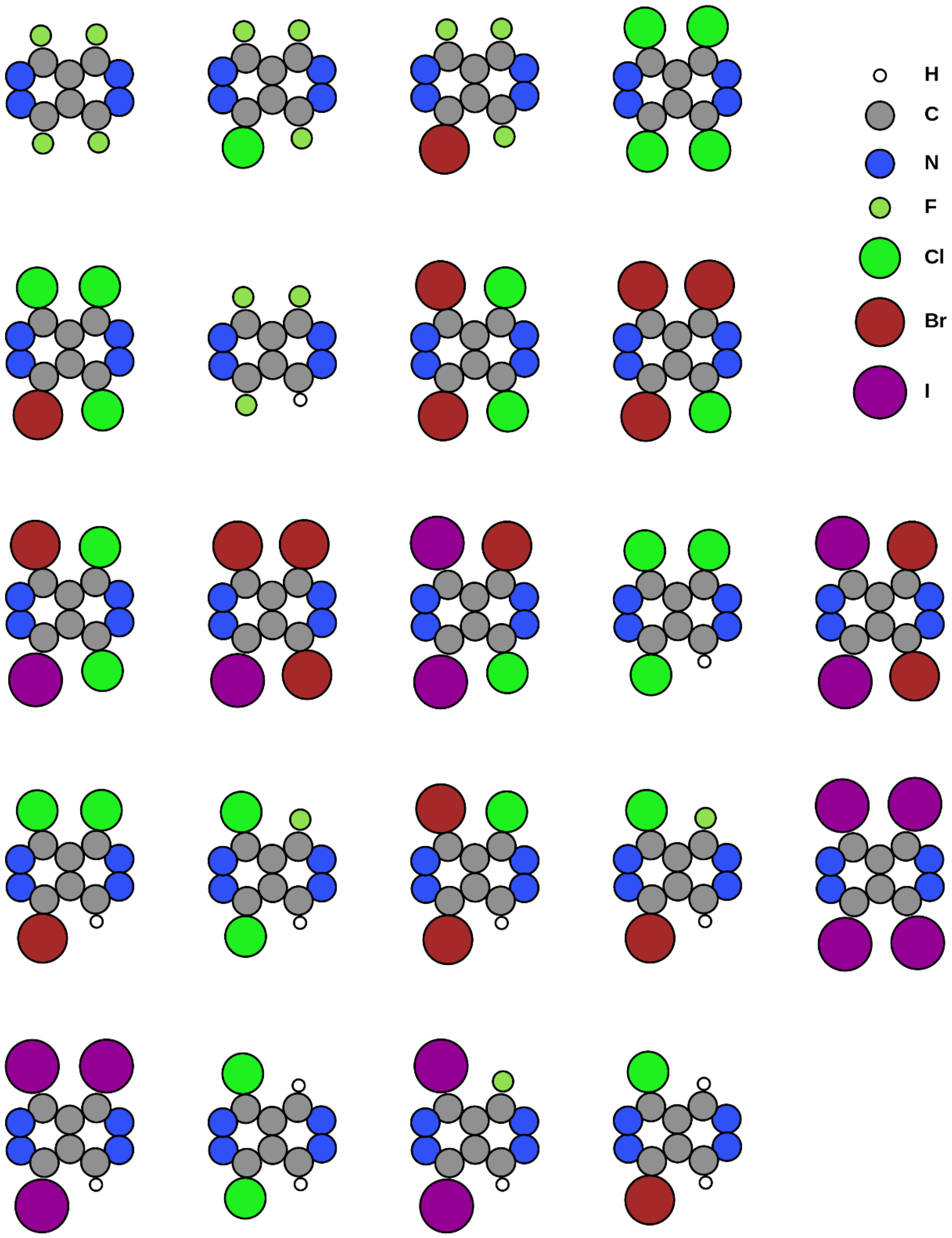

Figure S5: *Naphthalene-derived heteroaromatic molecules used to build metal-organic interface systems.*

Image Plane Positions for Substrates

As explained in the main text, substrate polarization effects can be modelled with a classical image potential $V_{\mathrm{im}(z)} = -\frac{1}{4(z-z_{\mathrm{im}})}$. The introduction of a test charge close to the surface must yield a response of the electronic density (due to screening of the test charge). The center of mass of this change must correspond to z_{im}.[3] We calculated the electronic density for the pristine surface and the surface with a perturbation of a positive charge of +0.01 electrons. In detail, we use a single atom with no basis functions and a modified core charge. This atom donates its "electron" (0.01 e^-) into the substrate yielding a positive charge. The test charge is put 7 Å and 10 Å above the surface. For both heights, the center of mass of the disturbance is calculated. Finally, the average is used.

We applied this method to all substrates used in this work. The resulting image plane positions are listed in Table ***S1***le S1.

Table S1: Image Plane Position for the Used Substrates.

substrate slab, (111) surface	image plane position / Å
Ag	2.278
Al	2.885
In	2.863
Mg	2.278
Na	4.241

Data Set Sanity and Alternative Approach

In order to check the fidelity of our data we attempted to find an expression for the work function change with an alternative set of parameters. The input parameters we used are listed in Table S2. We generated expressions by multiplying up to 5 input parameters (raised to the powers {-1, 1, 2}) with each other. Here, we also include parameters like the charge Q of the adsorbate. We note, however, that such a parameter is per se unphysical, because it is not an observable itself.

Table S2: Input Parameters Used to Generate Expressions for the Adsorption Energy

name	description
$h - z_{\mathrm{im}}$	adsorption height of the molecule w.r.t the image plane position
h	adsorption height of the molecule w.r.t the uppermost substrate layer
$\varepsilon_{\mathrm{HOMO}}$, $\varepsilon_{\mathrm{LUMO}}$	HOMO and LUMO orbital energy of adsorbate
$\partial\varepsilon_{\mathrm{LUMO}}/\,\partial n$	change of LUMO orbital energy w.r.t occupation
IP, EA	ionization potential and electron affinity of adsorbate
EA^{2nd}	second electron affinity
DOS(E_{F})	density of states of the pristine substrate at the Fermi energy
Φ_0	work function of the pristine substrate
Q	charge transferred from substrate to adsorbate (from Mulliken analysis)

We find

$$\Delta\Phi \propto Q(z - z_{\mathrm{im}}) \tag{2}$$

as best performing expression with an RMSE of 23 meV. This corresponds to the potential difference between the plates of plate capacitors with constant area (distance of the plates: z-z_{im}). Given the small RMSE and the very plausible expression, this result attests the sanity of our data.

Thus, the question arises if we could predict Q from input parameters of the interface constituents alone. Using the input quantities from Table S2 (except for Q itself) and allowing for products with up to 5 factors, all of which are powers of input parameters with the powers {-1, 1, 2}, we obtain

$$Q \propto \frac{\mathrm{EA} \cdot \mathrm{IP}^2}{h \cdot \mathrm{DOS}(E_\mathrm{F}) \cdot \Phi_0} \tag{3}$$

as best expression. Unfortunately, the expression is clearly unphysical, making this approach unsuccessful as well.

Choice of Non-Linear Mapping for the Construction of Expressions

Obviously, the choice of the non-linear mapping used to create expressions has great influence on the quality of the results of the symbolic regression. Unfortunately, as mentioned in the main paper, it is fundamentally impossible to proof if the "right" mathematical operations are included. We therefore tested the performance of several non-linear mappings. However, they all perform worse than the mapping specified in the main manuscript: F_i, F_j -> $F_i/(1+F_j)$.

For example, we tested the mapping F_i, F_j -> $F_i/(1+F_j^2)$ (using the input parameters as specified in the main manuscript, see Table S2). The resulting best performing expression is:

$$\Delta\Phi \propto (\varepsilon_\mathrm{LUMO} - E_\mathrm{F}) \cdot \frac{1}{1 + \left(\frac{\mathrm{EA}-\Phi}{h}\right)^2} \tag{4}$$

The result is very similar to the findings presented in the main paper, but performs slightly worse, with an RMSE of 47 meV.

We also tested more "exotic" mappings such as e.g. F_i, F_j -> log $|F_i/F_j|$. The best performing expression for this concrete example,

$$\Delta\Phi \propto (\varepsilon_\mathrm{LUMO} - E_\mathrm{F}) \cdot \frac{h - z_\mathrm{im}}{h}, \tag{5}$$

is also quite similar to our main result (expressions with log perform even worse) and exhibits an RMSE of 62 meV.

References

(1) Ouyang, R.; Curtarolo, S.; Ahmetcik, E.; Scheffler, M.; Ghiringhelli, L. M. SISSO: A Compressed-Sensing Method for Identifying the Best Low-Dimensional Descriptor in an Immensity of Offered Candidates. *Phys. Rev. Mater.* **2018**, *2* (8), 1–11. https://doi.org/10.1103/PhysRevMaterials.2.083802.

(2) Freedman, D.; Pisani, R.; Purves, R.; Adhikari, A. *Statistics*; WW Norton & Company New York, 2007.

(3) Lang, N. D.; Kohn, W. Theory of Metal Surfaces: Induced Surface Charge and Image Potential. *Phys. Rev. B* **1973**, *7* (8), 3541–3550. https://doi.org/10.1103/PhysRevB.7.3541.
